# 7-Tesla ultra-high field MRI of the parahippocampal cortex reveals evidence of common neurobiological mechanisms of major depressive disorder and neurotic personality traits

**DOI:** 10.1038/s41398-025-03435-y

**Published:** 2025-07-05

**Authors:** Dominik Nießen, Ravichandran Rajkumar, Dilsa Cemre Akkoc Altinok, Gereon Johannes Schnellbächer, Shukti Ramkiran, Jana Hagen, Nadim Jon Shah, Tanja Veselinović, Irene Neuner

**Affiliations:** 1 Department of Psychiatry, Psychotherapy and Psychosomatics, RWTH Aachen University, Aachen, Germany; 2 Institute of Neuroscience and Medicine 4, INM-4, Forschungszentrum Jülich GmbH, Jülich, Germany; 3 JARA-BRAIN – Translational Medicine, Aachen, Germany; 4 Institute of Neuroscience and Medicine 11, INM-11, Forschungszentrum Jülich, Jülich, Germany; 5 Department of Neurology, RWTH Aachen University, Aachen, Germany

**Keywords:** Depression, Psychology, Neuroscience

## Abstract

The parahippocampal cortex (PHC) is a highly interconnected region within the medial temporal lobe (MTL) and is essential in memory, emotion and cognition. According to the cognitive model of depression, dysfunctions in these processes constitute the pathophysiological foundation of major depressive disorder (MDD). Research suggests that human personality, and neuroticism in particular, plays an important role in the development and disease progression of MDD. Furthermore, extensive neuroimaging evidence indicates that neuroticism and depression share overlapping structural and functional correlates, potentially including the PHC. In a matched sample of 86 adults (43 MDD patients, 43 control participants, mean age 31.4 years, range 18–61 years, 40 female), PHC thickness was measured using structural MRI at an ultra-high field strength of 7 T and compared to the level of neuroticism as measured by the NEO-FFI scale. MDD patients exhibited significantly lower left hemispheric PHC thickness compared to healthy controls (*p*_*fdr*_ = 0.002, η^2^ = 0.119). Additionally, linear regression analysis revealed a significant association between neuroticism and PHC thickness within both hemispheres (L: *p*_*fdr*_ = 0.012, β = −0.414; R: *p*_*fdr*_ = 0.008, β = −0.512), with highly neurotic individuals displaying reduced cortical thickness. These findings suggest that, in combination with neuroticism, PHC thickness could serve as a potential biomarker of depression. Our results underscore the importance of multimodal assessments in MDD, potentially contributing to the foundation of individualised clinical decision-making and paving the way towards precision psychiatry.

## Introduction

Major depressive disorder (MDD) is a complex disease that can profoundly impact cognitive, emotional and social functioning. It is one of the leading causes of disability-adjusted life years (DALYs) worldwide, accounting for 49.4 million DALYs globally in 2020 [1]. In 2019, depressive disorders were ranked 13th among the leading causes of DALYs, with a global increase in prevalence of 63.7% since 1990 [2]. These figures underscore the importance of understanding the underlying mechanisms of MDD to improve its prevention, diagnosis and treatment.

Recent years have seen the emergence of translational sciences in psychiatry, aiming to identify quantifiable biological explanations that complement established theories of MDD, such as the cognitive model of depression [3, 4]. Translational neuroimaging, in particular, has provided significant insight into the underlying factors contributing to the development of MDD [5–8]. Advances in neuroimaging technologies have also facilitated the exploration of the neurobiological basis of personality traits, which are central determinants in the development of depression and other mental illnesses [9–11].

The five-factor model of personality (“Big Five”) has been the most prominent framework in personality psychology since the late 20^th^ century [12]. It comprises five complementary, orthogonal dimensions: neuroticism, extraversion, openness to experience, conscientiousness and agreeableness, contrasted with emotional stability, introversion, closed-mindedness, impulsivity and antagonism [12]. In the context of psychiatric disorders, neuroticism is of particular interest as it is a well-known risk factor for psychopathologies such as MDD, post-traumatic stress disorder (PTSD) and anxiety disorders [9–11, 13]. High neuroticism is characterised by negative affect, increased rumination and heightened emotional reactivity towards adverse events, all of which are also key features of MDD [14, 15]. In patients with MDD, neuroticism has been associated with greater symptom severity, prolonged recovery, low therapeutic success and a higher likelihood of relapse [11, 16–18].

The strong association between neuroticism and MDD has been further evidenced by genetic studies. Okbay and colleagues identified multiple common gene loci associated with both neuroticism and depressive symptoms, clearly demonstrating a biological basis for the relationship between neuroticism and MDD [19]. However, the common neurobiological basis of MDD and neuroticism remains poorly understood. Neuroimaging studies have revealed overlapping correlates of depression and neuroticism, with common neuroanatomical and neurofunctional peculiarities observed in structures within the limbic system, the medial temporal lobe (MTL) memory system, the frontal cortex and the default mode network (DMN) [6, 8, 20–22].

The parahippocampal cortex (PHC), a central hub embedded in the above-named brain systems, is involved in multiple cognitive processes that are often disrupted in MDD, including episodic, associative and source memory encoding and recollection, contextual processing, scene perception and emotional regulation [23–27]. Disturbances in these functions align with the cognitive model of depression, which postulates distorted information processing, such as negative interpretations of sensory information, selective attention towards negative aspects of information and the exclusion of positive aspects, resulting in heightened negative experiences and rumination [3]. This theoretical model is strongly backed by evidence from a multitude of studies [28–30] suggesting that, at a neuronal level, the described cognitive phenomena are caused by predominant bottom-up processes, dominated by limbic system activity [3, 28], coupled with impaired top-down inhibition from higher-order cortical regions, a dynamic referred to as limbic–cortical dysregulation [3, 28, 31, 32]. This dysregulation leads to an unrestrained activation of the limbic system and related structures, causing negative affect, altered memory processing, increased self-referential processing and depressive rumination [28]. Within the brain systems involved in these dysfunctional cognitive processes, namely the limbic system, the MTL memory system and the DMN, the PHC serves as a connecting element, both functionally and anatomically [26, 33]. Therefore, the PHC may play a central role in both the pathophysiology of MDD and the neurobiological underpinnings of neuroticism. In fact, several elements of the cognitive model of depression, such as maladaptive rumination and decreased reward-related memory function, have been successfully narrowed down to a neurobiological level, revealing, amongst other things, the involvement of the PHC [34–38]. However, the available literature does not allow for a comprehensive understanding of how and to what extent the PHC might show alterations on a structural level in the context of MDD and neuroticism. Given the paucity of information in this regard, this study investigates the possible association between PHC morphology and neuroticism and aims to demonstrate a neuromorphological correlate of MDD within the PHC.

Since the PHC is of importance due to its interconnective role within related brain systems, cortical thickness was selected as a suitable marker to measure dendritic arborisation and neuropil, rather than the number of neurons within the respective cortical area [39], as it allows conclusions to be drawn about neuronal connections within the target area. Changes in cortical thickness may therefore indicate alterations within the neural circuitry of the respective region. In the PHC specifically, this could affect functions such as memory encoding and recollection, as well as associative processing and emotional regulation, which are known to be impaired in both neurotic personality profiles and MDD [21–27].

Ultra-high field (UHF) MRI (with a field strength of 7 T) was employed to ensure optimal estimation accuracy, as comparative studies have shown that the increased signal-to-noise ratio (SNR) afforded by 7 T UHF-MRI can reveal disease-related changes within the human brain that are not detectable at 3T [40]. With signal-to-noise ratio (SNR) showing a linear increase with field strength, UHF-MRI allows for more valid quantitative results when using structural images [41]. The higher quality signal translates into a better spatial resolution and a noticeably increased tissue contrast in images acquired at 7T [42]. Therefore, neighbouring brain regions can be differentiated more precisely, and sensitivity towards subtle changes in regional brain morphology is increased [42–44]. This is particularly beneficial for the surface-based anatomical image analysis performed in this study [44]. Moreover, cortical thickness estimation is more accurate at 7 T when compared to 3T [45]. Since the improved SNR is particularly evident in temporal and frontal regions, studying these areas using UHF-MRI is especially beneficial [40]. To the best of our knowledge, this study is the first to implement 7 T UHF-MRI to investigate PHC morphology in patients suffering from MDD.

## Materials and methods

### Participants

The study included a total of 86 participants (mean age 31.4 years, SD 10.7, range 18–61 years), comprising 43 patients diagnosed with MDD (mean age 31.9 years, SD 10.7, range 18–61 years) and 43 control participants (mean age 30.9 years, SD 10.7, range 18–56 years). Age and biological sex were matched between the two groups using the case-control matching from IBM SPSS Statistics for Windows, Version 27.0 (Armonk, NY: IBM Corp), allowing a maximum age difference of 5 years, resulting in 20 females and 23 males per group. There was no significant difference in age and sex between the groups, as assessed via the Mann-Whitney U test for age (z = 0.661, *p* = 0.509) and the chi-square test for sex (χ^2^ (1, N = 86) = 0, *p* = 1.00). Demographic data and participant distribution are reported in Table 1. The required sample size was determined using G*Power for Windows, Version 3.1.9.3 (Heinrich-Heine-University, Düsseldorf, Germany) [46] to ensure a power of 0.8 at a significance level of *p* = 0.05, assuming an effect size of Cohen’s *f*^2^ = 0.1 and taking the number of covariates into account. The calculation resulted in a required sample size of 72 participants for a multivariate analysis of covariance (MANCOVA) and 81 participants for a linear regression analysis.

**Table 1:** Descriptive statistics.

	Control group (*N* = 43)		MDD group (*N* = 43)	
	mean (SD)	range	mean (SD)	range
Demographic				
Age (years)	30.9 (10.7)	18.8–56.7	31.9 (10.7)	18.3–61.0
Sex	F = 20 (46.5%)		F = 20 (46.5%)	
	M = 23 (53.5%)		M = 23 (53.5%)	
NEO-FFI				
Neuroticism	1.35 (0.61)	0.33–2.75	2.77 (0.52)	1.42–3.83
Extraversion	2.45 (0.63)	0.25–3.50	1.57 (0.67)	0.25–3.67
Openness for experience	2.51 (0.56)	1.25–3.58	2.57 (0.55)	1.25–3.58
Agreeableness	2.85 (0.41)	1.50–3.92	2.62 (0.47)	1.50–3.42
Conscientiousness	3.02 (0.51)	1.75–3.92	2.31 (0.68)	1.17–3.67
PHC thickness (mm)				
Left	2.59 (0.20)	2.11–2.99	2.45 (0.19)	1.91–2.95
Right	2.73 (0.23)	2.04–3.17	2.68 (0.20)	2.18–3.21

*MDD* major depressive disorder, *NEO-FFI* NEO Five-Factor Inventory, *PHC* parahippocampal cortex.

Patients were recruited from the RWTH Aachen University Hospital, Department of Psychiatry, and were required to meet the ICD-10 and DSM-5 criteria for MDD. In addition to the main diagnosis, eight patients had a personality disorder as secondary diagnosis, three patients suffered from post-traumatic stress disorder (PTSD), two patients from alcohol abuse, one patient from social phobia, one patient from a somatoform disorder, one patient from trichotillomania and two patients had shown psychotic symptoms in the past. Control participants were recruited from the surrounding community and via online advertising, with eligibility requiring legal age, good health, and no history of psychiatric or neurological disorders. Subclinical psychiatric disorders were excluded using the Structured Clinical Interview for DSM-IV for psychiatric axis-I-disorders (SCID-I) [47]. Handedness was assessed using the Edinburgh Handedness Inventory (EHI) [48], and only right-handed participants were included. Contraindications for MRI were considered.

A subset of the patient and control group overlapped with a data set published in earlier studies focussing on different objectives and methodologies (Altinok et. al. 2021 [49] and Schnellbächer et. al. 2022 [50]). The study was conducted in accordance with the recommendations of the Declaration of Helsinki, and the study protocol was approved by the Ethics Committee of the Medical Faculty of RWTH Aachen University. Written and verbal informed consent was obtained from all participants. All volunteers received compensation for their participation based on the time spent participating in the study and their travel expenses.

### NEO five-factor inventory (NEO-FFI)

The personality trait neuroticism was assessed using the NEO-FFI, a widely recognised [51] and cross-culturally applicable [52] instrument for measuring the “Big Five” factor model of personality [53]. The self-report-based questionnaire assesses the “Big Five” complementary personality domains of neuroticism, extraversion, openness to experience, agreeableness and conscientiousness through 60 items. Participants are required to rate given statements about their personality or behaviour on a five-fold graded scale ranging from “strongly disagree” to “strongly agree”. In this case, a well-established German translation was used [54], which demonstrated appropriate validity and satisfactory retest reliability [54, 55]. The NEO-FFI assessment was conducted within one week of the MRI, and only the neuroticism scores were considered for statistical analysis.

### MRI acquisition

MRI data acquisition was performed at Forschungszentrum Jülich, Germany, using a 7 T Magnetom Terra scanner (Siemens Healthineers, Erlangen, Germany) equipped with a 1Tx 32Rx Head Coil 7 T Clinical from Nova Medical (Wilmongton, MA, USA). Structural images were obtained using a T1-weighted MP2RAGE sequence, which is a modification of the standard magnetization-prepared rapid gradient echo (MPRAGE) sequence. MP2RAGE improves anatomical contrast by combining two gradient echo images acquired with different inversion times (IT) and flip angles (FA), thereby reducing the influence of B1 field inhomogeneities and enhancing brain tissue differentiation [56]. The sequence provides improved T1-weighted contrast while minimizing proton density and T2* contrast contributions, leading to a more accurate delineation of brain structures. To optimize the signal-to-noise ratio (SNR) while maintaining high spatial resolution, acquisition parameters were chosen based on previous optimization studies [56–58]. Specifically, the first inversion image (INV1) was acquired with IT = 840 ms, FA = 4°, echo time (TE) = 1.99 ms, and repetition time (TR) = 4500 ms. The second inversion image (INV2) was acquired with IT = 2370 ms, FA = 5°, TE = 1.99 ms, and TR = 4500 ms. The image matrix was set to 320 mm × 300 mm, achieving a 0.75 mm isotropic resolution in 208 sagittal slices. These parameters were selected to balance the trade-off between T1 contrast and SNR while minimizing transmit field inhomogeneities, as described in previous studies [56, 58]. The final MP2RAGE unified image was generated using a division approach that minimizes sensitivity to reception bias fields, proton density contrast, and T2* contrast [56].

### Structural MR data preprocessing

First, the raw DICOM scans were converted into 3D T1-weighted Neuroimaging Informatics Technology Initiative (NIfTI) format using MRIcron software (https://www.nitrc.org/projects/mricron). All 3D T1-weighted images were visually inspected using FSL View software (https://fsl.fmrib.ox.ac.uk/fsl/fslwiki/FslView) to detect artefacts and tissue abnormalities and to ensure good image quality. Voxel-based morphometry (VBM) was performed using the Computational Anatomy Toolbox (CAT12.8; version 1907) preprocessing pipeline (https://www.neuro.uni-jena.de/cat/index.html#VBM), which is designed to work with the Statistical Parametric Mapping (SPM12) toolbox (https://www.fil.ion.ucl.ac.uk/spm/software/spm12/) [59] and MATLAB (version 9.7 (R2019b)). VBM preprocessing was performed with CAT12.8 using the default settings. Following spatial adaptive non-local means (SANLM) denoising [60] and correction for bias field inhomogeneities, the 3D T1-weighted structural images underwent SPM12 affine registration to a standard reference space. Unified segmentation [59], based on the standard Tissue Probability Map (TPM) provided by the SPM toolbox, was used to generate the starting estimates of grey matter (GM), white matter (WM) and cerebrospinal fluid (CF) for subsequent refined voxel-based preprocessing steps. The steps included skull stripping using the adaptive probability region-growing (APRG) method, brain parcellation (into both hemispheres, subcortical areas and the cerebellum), detection of WM hyperintensities, and local intensity transformation of all tissue classes. Afterwards, adaptive maximum a posteriori (AMAP) segmentation [61] and partial volume estimation were applied to each voxel [62] to account for the partial volume effect. Finally, the tissue segments were spatially normalised to the same stereotactic MNI152NLin2009cAsym reference space using DARTEL [63].

Surface-based processing was performed utilising the CAT12.8 toolbox in default settings. A projection-based thickness (PBT) approach, which has been shown to be less error-prone when compared to other methods, was used to simultaneously compute cortical thickness values and reconstruct the central surface [64]. Topology defects in the surfaces generated with the PBT were corrected using spherical harmonics [65] followed by surface refinement. The resulting central surface mesh was then spatially registered to the ‘FsAverage’ template of Freesurfer individually for each participant using a spherical mapping with minimal distortions [66]. Lastly, the local cortical thickness values generated with the PBT were transferred onto the ‘FsAverage’ template.

Parcellation of the cortical surface was performed using the ‘Desikan-Killiany’ (DK-40) cortical atlas [67]. The DK-40 atlas is widely used, and the resulting estimates of cortical thickness have been shown to agree well with post-mortem histologic measures of cortical thickness within corresponding regions [68]. The cortical thickness of the parahippocampus was computed separately for both hemispheres in the native space of each participant, and the mean thickness values within the region were considered for further analysis.

Image and preprocessing quality were ensured through the automated scoring system provided by the CAT12.8 toolbox [69]. The mean and standard deviations of the overall weighted image quality before preprocessing were 87.9 ± 3.3% across all participants, taking resolution, noise and bias into account. The mean Euler number, indicating the number of topology defects of the extracted surface, was 66 ± 34 and the mean defect area was 1.9 ± 1.1% across all participants. Additionally, GM segments from each participant were overlaid onto the structural image and visually for any non-brain structures.

### Statistical analysis

Statistical analysis was performed using SPSS Version 27.0. The Levene’s test was used to check the assumption of homogeneity of variances, and the normal distribution of residues was assessed with the Shapiro-Wilk test for all variables of interest. Intergroup differences of clinical and demographic data exhibiting a normal distribution were analysed using an independent samples t-test (neuroticism), while non-normally distributed parameters were analysed using the Mann-Whitney U tests (age). Nominal variables were evaluated using a chi-square test (sex). To assess the consistency with previous studies and the representativeness of the sample, correlations between neuroticism and age were investigated separately in both groups using a Pearson correlation, and gender-dependent differences in the neuroticism scores were assessed using an independent samples t-test with a significance level set at *p* < 0.05.

Differences in PHC thickness between MDD patients and the healthy control group were analysed using a multivariate analysis of covariance (MANCOVA). The assumption of equivalence of covariance matrices was checked with Box’s M test and the intercorrelation of left and right hemispheric PHC thickness was verified as acceptable using a Pearson correlation with *p* < 0.001, r = 0.60. Group affiliation was chosen as the independent variable, and left and right hemispheric PHC thickness was the dependent variable. Age and sex were included in the statistical model as covariates. Next, a linear regression analysis was conducted to investigate the relationship between PHC thickness and the NEO-FFI neuroticism score further. Parahippocampal cortical thickness was defined as the dependent variable, and the neuroticism score was the predictor variable. Results were adjusted for age, sex and group affiliation as confounding factors. The significance level was set at *p* < 0.05 and the Benjamini-Hochberg method was used to account for multiple testing. FDR-corrected *p*-values were calculated for all final analyses and labelled *p*_*fdr*_. Effect sizes were determined via partial η^2^ and standardised beta coefficient, respectively.

## Results

### NEO-FFI

Results of the NEO-FFI are reported in Table 1. The calculated values for the healthy control sample were consistent with findings from larger, representative samples [55]. In order to assess the consistency of the 12 individual items with the total NEO-FFI neuroticism score within our sample and to exclude a high proportion of random answers, Crohnbach’s Alpha (α) was calculated, resulting in a value of α = 0.919. Scale means for neuroticism were 2.77 (SD = 0.52) in the MDD group and 1.35 (SD = 0.61) in the healthy control group. MDD patients exhibited significantly higher NEO-FFI neuroticism scores compared to healthy controls, t(84) = −11.67, *p* < 0.001. Consistent with previous findings, younger age was significantly associated with higher neuroticism scores in the healthy control group (r(41) = −0.31, *p* = 0.041) [70]. However, this effect could not be observed in the MDD group (*p* = 0.668) (Supplementary Table 1).

### Parahippocampal cortical thickness

**Fig. 1:**
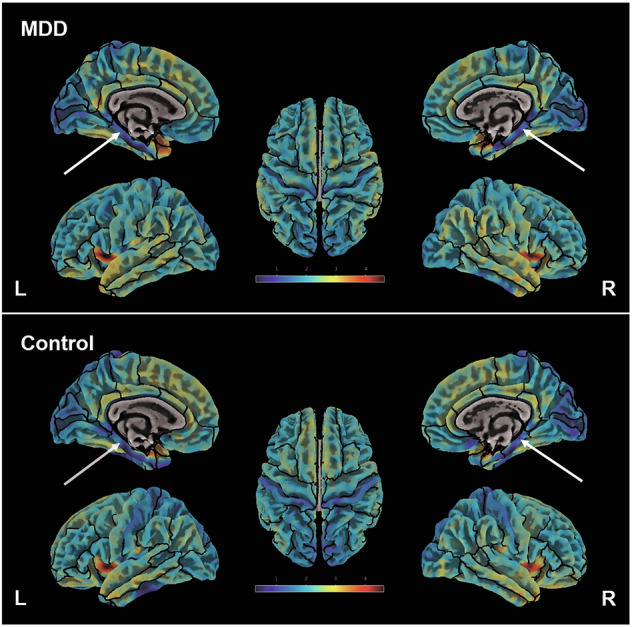
Cortical thickness map of a representative control participant and a patient with MDD. A medial and lateral view of the left hemisphere are illustrated on the left side of the image. In the centre is a top view. A corresponding view of the right hemisphere is shown on the right. Boundaries of the Desikan-Killiany DK-40 atlas are outlined in black. The PHC is highlighted by an arrow. Significant intergroup differences were found in the left hemispheric PHC thickness. Neuroticism was correlated negatively with both left and right hemispheric PHC thickness; MDD major depressive disorder, PHC parahippocampal cortex.

Cortical thickness maps are illustrated in Fig. 1. The comparison of the MRI measurements delineated significant results in support of our hypothesis that PHC thickness differs between groups.

**Fig. 2:**
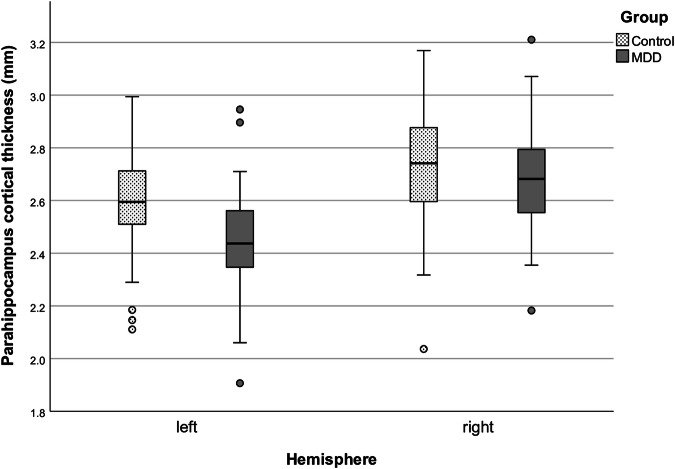
Boxplots comparing the PHC thickness of control participants and depression patients illustrated separately for both hemispheres; PHC parahippocampal cortex, MDD major depressive disorder.

The MANCOVA revealed a significant effect of the group affiliation on the combination of left and right parahippocampal cortical thickness when adjusted for age and sex (Wilk’s Λ = 0.87, (F(2,81) = 6.18, *p* = 0.003, η^2^ = 0.132; Supplementary Table 2). In comparison to the healthy control group, left hemispheric PHC thickness was lower in MDD patients (F(1,82) = 11.12, *p*_*fdr*_ = 0.002, R^2^_adjusted_ = 0.145, η^2^ = 0.119; Fig. 2; Supplementary Table 3). However, the difference in right hemispheric PHC thickness between groups did not meet the threshold of significance (*p*_*fdr*_ = 0.328). PHC thickness values are reported in Table 1.

### Regression analysis

**Fig. 3:**
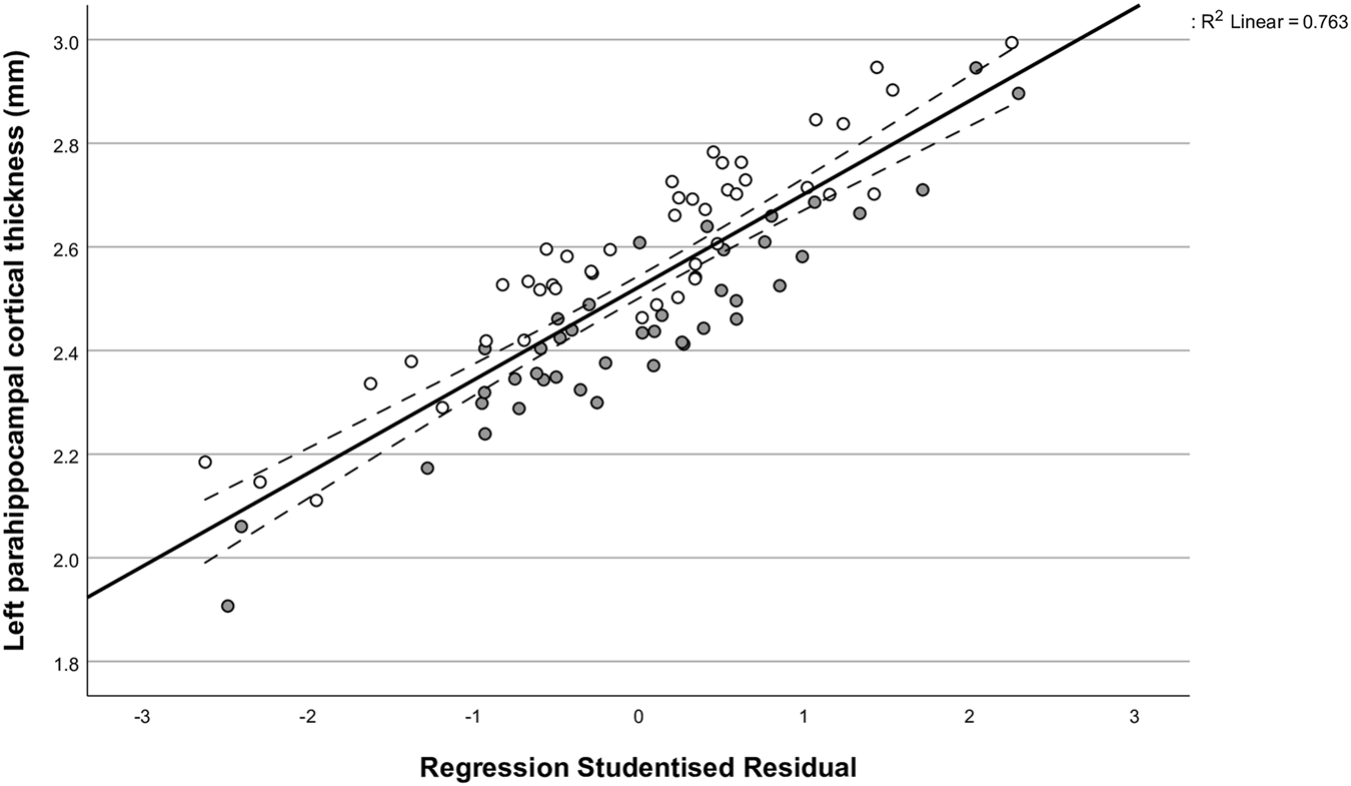
Linear regression plot alongside the 95% confidence interval illustrating the association between the left PHC thickness and the NEO-FFI neuroticism score after correcting for age, sex and group affiliation (*p*_*fdr*_ = 0.012, β = −0.414). Individual data points of depression patients are illustrated in grey, and those of control participants in white. The fitted regression model was: Left PHC cortical thickness (mm) = 2.902 – 0.070 (sex) – 0.005 (age) – 0.003 (group) – 0.094 (NEO-FFI neuroticism) (R^2^_adjusted_ = 0.200, F(4, 81) = 6.30, *p* < 0.001); PHC parahippocampal cortex, MDD major depressive disorder.

**Fig. 4:**
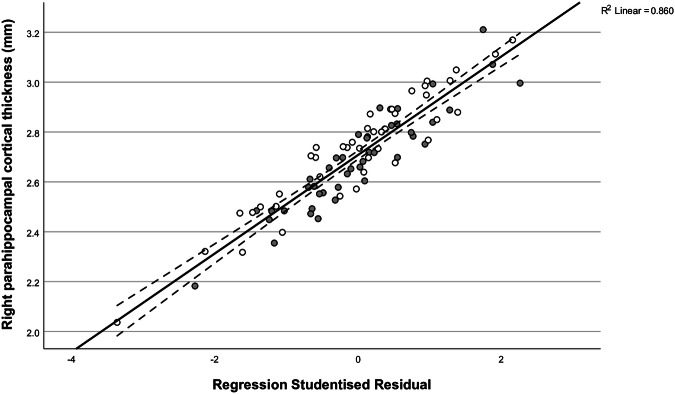
Linear regression plot alongside the 95% confidence interval illustrating the association between the right PHC thickness and the NEO-FFI neuroticism score after correcting for age, sex and group affiliation (*p*_*fdr*_ = 0.008, β = −0.512). Individual data points of depression patients are illustrated in grey, and those of control participants in white. The fitted regression model was: Right PHC cortical thickness (mm) = 3.060 – 0.035 (sex) – 0.005 (age) – 0.128 (group) – 0.121 (NEO-FFI neuroticism) (R^2^_adjusted_ = 0.098, F(4, 81) = 3.31, *p* = 0.014); PHC parahippocampal cortex, MDD major depressive disorder.

The next step of our analysis aimed to investigate a possible relationship between differences in PHC thickness and neuroticism as measured by the NEO-FFI. Linear regression analysis revealed a significant association between the cortical thickness of both the left and right hemispheric parahippocampus and the NEO-FFI neuroticism score after correcting for age, sex and group affiliation (L: *p*_*fdr*_ = 0.012, β = −0.414; R: *p*_*fdr*_ = 0.008, β = −0.512; Supplementary Table 4). The fitted regression models were: L: PHC cortical thickness (mm) = 2.902 – 0.070 (sex) – 0.005 (age) – 0.003 (group) – 0.094 (NEO-FFI neuroticism) (R^2^_adjusted_ = 0.200, F(4, 81) = 6.30, *p* < 0.001) and R: PHC cortical thickness (mm) = 3.060 – 0.035 (sex) – 0.005 (age) – 0.128 (group) – 0.121 (NEO-FFI neuroticism) (R^2^_adjusted_ = 0.098, F(4, 81) = 3.31, *p* = 0.014). Regression plots are illustrated in Fig. 3 and Fig. 4.

## Discussion

Taking advantage of the enhanced signal provided by 7T-UHF-MRI, our analysis confirmed a significant reduction in PHC thickness in MDD patients compared to control participants. Furthermore, PHC thickness decreased with higher NEO-FFI neuroticism scores. These findings highlight the involvement of the PHC in the pathophysiology of MDD and suggest a tangible neurobiological link between neuroticism and MDD. Furthermore, in the context of available literature, the results also support the cognitive model of depression, which proposes that negative cognitive biases contribute to depressive symptomology.

Our results are consistent with previous neuroimaging studies reporting reduced PHC GM volume in patients with depression [38, 71, 72]. The pathophysiological significance of the PHC in depression can be derived from its integration into several brain systems. These systems are, most importantly, the MTL memory system, the DMN and the limbic system [24, 26, 33, 73]. This system affiliation suggests that the PHC may serve as a central hub that is involved in interlocking emotional processing as facilitated by the limbic system, with episodic, source and especially associative memory as processed by the MTL memory system [26]. Furthermore, the PHC was identified as a connecting element between the MTL memory system and the DMN [33]. In summary, the PHC likely plays an important role in the interplay of associative memory, emotion and mind-wandering while at rest [26]. The reduced thickness of the PHC in the MDD patients observed in our study may be indicative of a disturbance in this intricate interplay, leading to biased associative memory processing, negative affect and depressive rumination - as proposed by the cognitive model of depression.

Additionally, decreased GM volume in the PHC has been associated with ruminative tendencies in MDD [38]. A comparison of fMRI activity patterns between nonmedicated patients with MDD and healthy controls revealed reduced PHC activity during an associative memory task. This finding suggests that diminished associative processing within the PHC contributes to depressive rumination [38]. In another structural MRI study, the authors were able to predict dysfunctional cognition in the form of automatic thoughts through the interaction of neuroticism and PHC GM volume [74]. Furthermore, neuroticism moderated the relationship between depressive symptoms and PHC GM volume [74]. However, in their study, higher PHC GM volume correlated positively with stronger negative automatic thoughts, and, in contrast to our results, the authors could not prove an association between neuroticism and PHC GM volume [74].

On a pathophysiological level, our findings align well with the current state of knowledge on emotional regulation and memory processing. Research suggests that emotions, which are generated in the limbic system, are regulated by top-down signalling from the prefrontal cortex [31]. The majority of afferent cortical fibres enter the limbic lobe via the perforant pathway within the PHC region, ultimately projecting into the hippocampus [73, 75, 76]. A reduced PHC thickness may represent a reduction in such regulatory cortical afferences, leading to the impairment of top-down inhibition of processes within the limbic system. On a symptom level, this limbic-cortical dysregulation may facilitate negative affect and rumination [28]. This hypothesis is also supported by findings of reduced functional connectivity between cortical structures and hippocampal formation in resting state fMRI data from MDD patients [77]. Similarly, signs of limbic-cortical dysregulation can also be observed in the fMRI of highly neurotic individuals, as indicated by a reduced connectivity between the amygdala and the anterior cingulate cortex (ACC) [78]. Our observation of reduced PHC thickness in neuroticism, which is first and foremost a marker for negative affect [51], further underscores the involvement of the PHC in regulating emotions. In summary, structural alterations within the PHC may indicate emotional dysregulation and, therefore, represent an early manifestation or predisposing factor to MDD. This is in line with findings relating to reduced PHC GM volume in subclinical depression [71].

As elaborated above, a reduced PHC thickness may be indicative of a disconnection between the hippocampal formation and the cortex. This may also have implications in the context of altered memory processing, as suggested by the cognitive model of depression. Research on anatomical connectivity shows that PHC mediates both the afferent and efferent connections of the hippocampus as they are passing through [33, 76]. Therefore, memory processes are likely highly dependent on the integrity of the PHC. Indeed, animal studies have shown that isolated injury to the PHC leads to the heavy impairment of memory function [75]. Furthermore, diffusion tensor imaging data of the human brain shows a decreased signal within the perforant pathway in association with reduced age-related memory performance [79]. These findings imply that a rarefication of connections within the PHC, as indicated by reduced cortical thickness in our MDD group, may also lead to functional changes within the memory system. In a source memory task, patients suffering from MDD showed weaker activation of the right PHC when accessing memory for a reward source when compared to a healthy control group, thus supporting the theory of altered memory encoding for positive stimuli within the PHC in depression [34]. Furthermore, reduced functional connectivity between reward-related areas in the medial orbitofrontal cortex (mOFC) with the parahippocampal gyrus was demonstrated in MDD, similarly indicating a dysfunction in reward-related memory systems [35]. Simultaneously, the PHC may also be involved in enhancing memory encoding for negative stimuli, as PET data shows a stronger interaction between the PHC and the amygdala during the encoding of emotionally negative film clips as opposed to neutral film clips [80]. Further to providing a tangible, neurobiological explanation for cognitive biases when processing emotionally connotated memories in MDD, these findings give an incentive to investigate the PHC in the context of depressive cognitive disorders, also referred to as pseudodementia. This phenomenon is associated with the severity of depression and clinically manifests as deficits in explicit memory that resemble dementia [81]. A neuronal basis of this entity within the MTL memory system has previously been suspected [81, 82], and further investigation of the PHC may prove fruitful in this context.

Finally, our finding of reduced PHC thickness in MDD supports a growing body of evidence that the PHC may be an important structural target in the treatment of depression. Regarding the concrete underlying neuronal mechanisms of antidepressant medication, there is evidence of neuronal reconstitution within affected brain regions through both neuroplasticity and neurogenesis [83, 84]. Concretely, this is believed to be facilitated by the release of neurotrophic factors, such as BDNF, in the hippocampal formation and the cerebral cortex [84, 85]. While findings from animal models show a reduction in neural cell proliferation, neurogenesis, and synaptic function in the hippocampal formation of rats with learned helplessness, they also show the formation of new dendritic connections in the same area when administering antidepressant drugs [86, 87]. In this context, several studies point concretely towards the PHC. Firstly, in a study conducted by An and colleagues, an eight-week treatment with escitalopram led to an increase in short-distance regional functional connectivity within the parahippocampus region in MDD patients, suggesting that the parahippocampus may, in fact, exhibit structural reorganisation under antidepressant medication [88]. Secondly, Paolini and colleagues showed worse treatment outcomes in MDD patients with reduced hippocampal and parahippcoampal GM volume [89]. In addition to the hippocampus, this also suggests the PHC as an important site of action for the previously mentioned neurotrophic effect of antidepressant drugs [89]. In conclusion, a reduced hippocampal and parahippocampal GM volume may indicate a lower density of drug targets or lower regenerative potential under antidepressant medication due to a lasting loss of neurons and glia. Interestingly, in our sample, we observed a dependency of the reduction of PHC cortical thickness on the NEO-FFI neuroticism score. This very much aligns the findings of Paolini with observations made by Kudo and colleagues, who demonstrated that neurotic personality traits would, in fact, also predict worse treatment outcomes in MDD [17]. In conclusion, reduced PHC thickness and higher neuroticism may, therefore, conjointly serve as markers to detect treatment-resistant depression and enable appropriate treatment measures to be taken at an early stage.

We have argued that dysfunctional cognition is crucial for the development and maintenance of depression. In patients suffering from MDD, these cognitive processes may be modified through pharmacotherapy [90]. In one study, a single dose of reboxetine was able to decrease response latency and improve memory for positive words in depressed patients performing an emotional processing task, suggesting that antidepressant medication may have a positive effect on dysfunctional cognition [91]. Interestingly, it could be shown that the positive effects of pharmacotherapy on dysfunctional cognition are not limited to patients suffering from MDD but can also be extended to highly neurotic individuals. Di Simplicio and colleagues showed reduced DMN activation as a therapeutic effect of citalopram in highly neurotic individuals during a negative self-referential word categorisation task. This suggests that citalopram may positively influence specific neural dysfunctions that lead to negative cognitive biases [92]. These results also show that there might be an incentive for early initiation of therapy and administration of antidepressant medication in subclinical depression based on parameters like the NEO-FFI neuroticism score. Moreover, there are reassuring findings showing reduced neuroticism under pharmacotherapy in conjunction with an improvement in depressive symptoms [93]. This observation underscores the supposition that neuroticism can be positively influenced to mitigate its impact on symptom severity, recovery and relapse in MDD.

Considering the observed association between increased neuroticism and reduced PHC thickness, the co-occurrence of both traits in an individual may indicate a predisposition to the development of MDD or to treatment resistance in its course. In such a situation, early or even preventive treatment, including the administration of antidepressants, could conceivably prevent the occurrence of MDD or contribute to a more favourable outcome by stimulating neuronal processes within the hippocampal formation of at-risk individuals. However, further longitudinal studies are needed to explore this hypothesis.

### Conclusion

To summarise, our results confirm a reduced thickness of the PHC in patients suffering from MDD. In addition, we found that reduced PHC thickness is associated with more pronounced neurotic personality traits. Our findings indicate that structural alterations within the PHC may play an important role in enabling dysfunctional cognitive processes, as observed in MDD and neurotic personality profiles. Moreover, in conjunction with neuroticism, a reduction in PCH thickness could serve as a useful biomarker for increased risk or the course of depression. Thus, our study results support the idea that a multimodal approach to the investigation of MDD using psychological assessments and the examination of brain morphology as complementary tools may contribute to a better understanding of the disease. We consider ultra-high field MRI to be particularly promising for gaining further insight into the pathophysiology of MDD. In the future, a multimodal assessment of patients may also contribute to a more comprehensive basis for individualised decision-making in a clinical context. This may ultimately prove beneficial for prevention, early detection, treatment selection, and prognosis assessment and pave the way towards precision psychiatry.

## Supplementary information

Supplementary material

## Data Availability

Source data can be made available through contact with the principal investigator, Prof. Dr. Neuner.
