# Supplementary material for: 7-Tesla ultra-high field MRI of the parahippocampal cortex reveals evidence of common neurobiological mechanisms of major depressive disorder and neurotic personality traits
Source: Transl Psychiatry. 2025 Jul 5;15:227. doi: 10.1038/s41398-025-03435-y (PMC12228691; doi:10.1038/s41398-025-03435-y)
Supplement: Supplementary file 1 — Supplementary material [file 41398_2025_3435_MOESM1_ESM.docx]

# SUPPLEMENTARY INFORMATION

Supplementary Table 1: Independent samples t-test of neuroticism between genders and Pearson correlation of neuroticism and age, results reported separately for MDD and control participants

*MDD* major depressive disorder

Supplementary Table 2: MANCOVA examining the effects of group affiliation, sex and age on the left and right hemispheric parahippocampal cortical thickness

Supplementary Table 3: Test of between-subject effects for left and right hemispheric parahippocampal cortical thickness

a R Squared = .175 (Adjusted R Squared = .145), b R Squared = .045 (Adjusted R Squared = .010), *PHC* parahippocampal cortex

Supplementary Table 4: Linear regression analyses of left and right hemispheric parahippocampal cortical thickness

Fitted regression models: L: PHC cortical thickness (mm) = 2.902 – 0.070 (sex) – 0.003 (group) – 0.005 (age) – 0.094 (NEO-FFI neuroticism),

R: PHC cortical thickness (mm) = 3.060 – 0.035 (sex) – 0.005 (age) + 0.128 (group) – 0.121 (NEO-FFI neuroticism), *PHC* parahippocampal cortex
